# Synthesis of Endohedral Metallofullerene Glycoconjugates by Carbene Addition

**DOI:** 10.3390/molecules16119495

**Published:** 2011-11-14

**Authors:** Michio Yamada, Chika I. Someya, Tsukasa Nakahodo, Yutaka Maeda, Takahiro Tsuchiya, Takeshi Akasaka

**Affiliations:** 1 Department of Chemistry, Tokyo Gakugei University, Koganei, Tokyo 184-8501, Japan; 2 Life Science Center of Tsukuba Advanced Research Alliance, University of Tsukuba, Tsukuba, Ibaraki 305-8577, Japan

**Keywords:** chemical functionalization, La_2_@*I_h_*-C_80_, carbohydrate, diazirine, glycoconjugate

## Abstract

Endohedral metallofullerene glycoconjugates were synthesized under mild conditions by carbene addition using appropriate glycosylidene-derived diazirine with La_2_@*I_h_*-C_80_. NMR spectroscopic studies revealed that the glycoconjugate consists of two diastereomers of [6,6]-open mono-adducts. The electronic properties were characterized using Vis/NIR absorption spectroscopy and electrochemical measurements. This study demonstrates that glycosylidene carbene is useful to incorporate carbohydrate moieties onto endohedral metallofullerene surfaces.

## 1. Introduction

Recent developments in the chemistry of endohedral metallofullerenes (EMFs) [1,2,3,4] have sparked increasing interest in their biochemical and medicinal applications. Particularly, great interest has been directed toward development of magnetic resonance imaging (MRI) contrast and therapeutic agents based on EMF scaffolds [5,6,7,8,9,10,11,12,13,14,15,16,17,18]. Robust fullerene cages protect encaged metal ions from any potential metabolic process, therefore, EMFs can act as nanocarriers with no release of toxic metal ions. In this context, chemical derivatization of EMFs to introduce functions such as solubility, permeability, and site-specific recognition ability is indispensable. To date, however, exohedral chemical functionalization of EMFs has remained limited to introduction of groups that do not introduce additional features because of the different reactivity from that of C_60_ [19].

We explored the reactivity of EMFs and found that reactions of EMFs with electrophilic carbenes proceed smoothly to afford the formation of corresponding EMF derivatives quantitatively [20,21,22,23]. These results encouraged us to synthesize functionalized EMF conjugates by such carbene addition. A carbohydrate moiety was selected as a functional group for this study because carbohydrate–protein interactions are encountered in many biological events. In addition, deprotection of the carbohydrate residues could potentially generate ambiphilic EMFs, leading to biochemical and pharmacological investigations [24,25,26,27,28,29,30,31,32,33]. This report describes the synthesis of endohedral metallofullerene glycoconjugates by carbene addition for the first time.

## 2. Results and Discussion

We adopted La_2_@*I_h_*-C_80_ as a representative EMF scaffold because: (1) La_2_@*I_h_*-C_80_ has icosahedral symmetry, which enables reduction of the number of possible isomers of the adducts; (2) its diamagnetic character enables characterization of the molecular structure using NMR spectroscopy; and (3) among lanthanum EMFs La_2_@*I_h_*-C_80_ is obtainable in the second highest yield by direct-current arc-discharge process, whereas La@*C*_2*v*_-C_82_ is the main product.

**Scheme 1:**
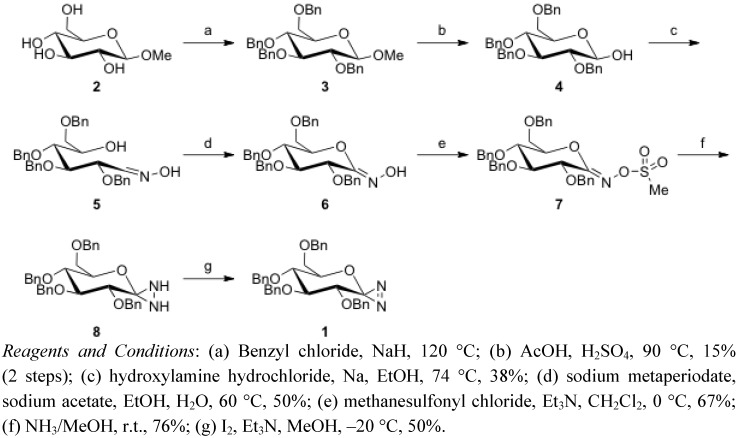
Synthesis of glycosylidene diazirine **1**.

Glycosylidene-derived diazirine **1** was synthesized according to reports in the literature by Vasella *et al.*, as summarized in Scheme 1 [34,35].

Reaction of commercially available methyl-α-D-glucopyranoside **2** with benzyl chloride in the presence of sodium hydride yielded *O*-benzyl derivative **3** [36]. The pyranoside anomeric hydroxyl group was deprotected with sulfuric acid to give 2,3,4,6-tetra-*O*-benzyl-D-glucopyranose (**4**) [37]. This product was condensed with hydroxylamine hydrochloride in the presence of sodium to provide open-chain oxime**5** as a mixture of stereoisomers [38]. Oxidative cyclization of **5** with sodium metaperiodate provided the desired ring-closed material **6**. Treating hydroximinolactone **6** with methanesulfonyl chloride under basic conditions yielded the corresponding methanesulfonate **7**. Reaction of **7** with ammonia yielded diaziridine **8**, which was subsequently oxidized by iodine to afford diazirine **1** [34,35].

**Scheme 2:**
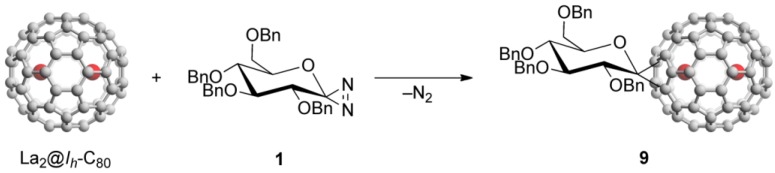
Reaction of La_2_@*I_h_*-C_80_ with diazirine **1**.

Endohedral metallofullerene glycoconjugate was synthesized by the reaction of La_2_@*I_h_*-C_80_ with **1**, as shown in Scheme 2.

**Figure 1:**
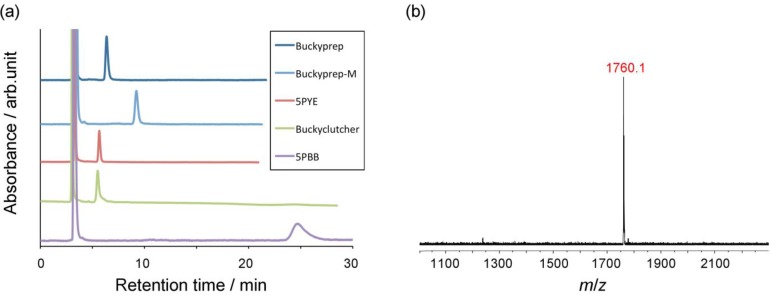
(a) HPLC traces of purified **9**. Conditions: 4.6 mm × 250 mm i.d. columns; eluent, toluene 1.0 mL/min; (b) Negative-mode MALDI-TOF mass spectrum of **9**. 9-Nitroanthracene was used as matrix.

Compound **1** easily generates the corresponding glycosilydene carbene at room temperature, which is allowed to react smoothly with La_2_@*I_h_*-C_80_ to afford the formation of La_2_@*I_h_*-C_80_ glycoconjugate **9**. The HPLC analysis of the reaction mixture suggested that **9** was formed predominantly. The mixture was subjected to HPLC separation to purify **9**. As shown in Figure 1(a), the HPLC profiles of the purified **9** using different columns exhibited single peaks.

**Figure 2:**
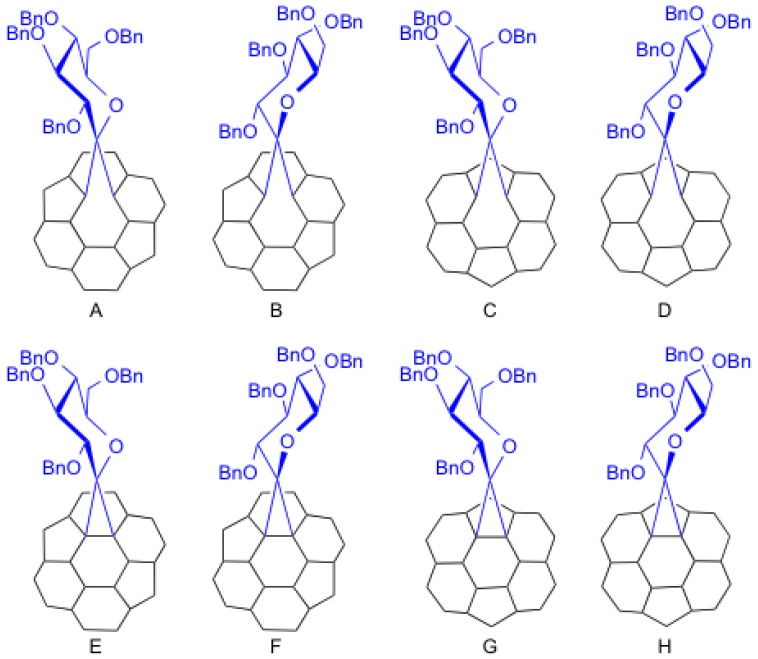
Partial structures of the eight possible isomers A–H.

The matrix-assisted laser desorption/ionization time-of-flight (MALDI-TOF) mass spectrum of **9** clearly displayed the expected molecular ion peak at *m*/*z* 1760.1 (calcd. for C_114_H_34_O_5_La_2_: 1760.05), as shown in Figure 2(b). In addition, circular dichroism (CD) bands were observed at 390–550 nm, confirming that the chiral glucopyranose moiety was introduced successfully onto the EMF surface (see Figure S1 in the Supporting Information). The solubility of **9** in common organic solvents is higher than that of La_2_@*I_h_*-C_80_(Ad) (Ad = adamantylidene), presumably because of the introduction of polarity with the sugar-like structure.

Theoretically, eight possible isomers (A–H) exist for conjugate **9**, as shown in Figure 2. All isomers have *C*_1_ symmetry. In isomers A, B, E, and F, the addition took place at a C–C bond that bisects two hexagonal rings (so-called [6,6]-addition). In C, D, G, and H, the addition took place at a C–C bond that bisects hexagonal and pentagonal rings (so-called [5,6]-addition). In addition, the C–C bond was cleaved by the addition in isomers A–D (so-called open form). The addition yielded a cyclopropane ring on the cage in isomers E–F (so-called closed form).

**Figure 3:**
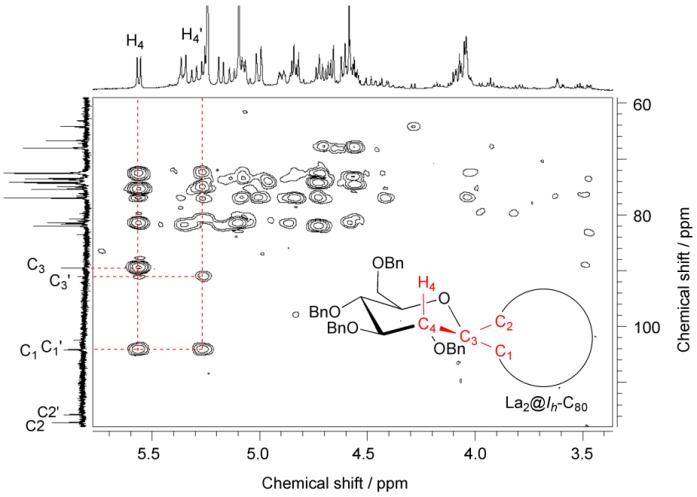
500 MHz HMBC NMR spectrum of **9** in CD_2_Cl_2_/CS_2_ (v/v 1:3) at 303 K. Inset shows the schematic structure of **9**.

NMR spectroscopic studies revealed that **9** contains two inseparable diastereomers in a ratio of ca. 1:1 because two sets of signals were observed in the ^1^H- and ^13^C-NMR spectra although a single signal was observed in the ^139^La-NMR spectrum (see Figures S2,3 in the Supporting Information). In fact, 117 quaternary carbon signals appeared in the ^13^C-NMR spectrum, which are associated with the sp^2^ cage carbon atoms and benzene rings. In addition, two ^13^C signals at 91.09 and 89.51 ppm are attributed to spiro carbon atoms (designated as C_3_ and C_3_′) on the glycosilydene moiety, indicating the presence of two isomers. The ^13^C signals of the cage carbon atoms bonded to the glycosilydene moiety (designated as C_1_ and C_1_’) appeared at 104.16 and 104.11 ppm. In fact, the two signals are correlated with the axial proton atoms (designated as H_4_ and H_4_′) on the glycosilydene ring in the HMBC NMR spectrum as shown in Figure 3. Observations also indicate that the diastereomers possess not closed forms but open forms because C_1_ and C_1_’ carbon atoms can be regarded as sp^2^-carbon atoms. In contrast, correlation between H_4_ and the other carbon atoms designated as C_2_ (or C_2_′) at 117.16 and 115.75 ppm in Figure 3, was not observed. 

**Figure 4:**
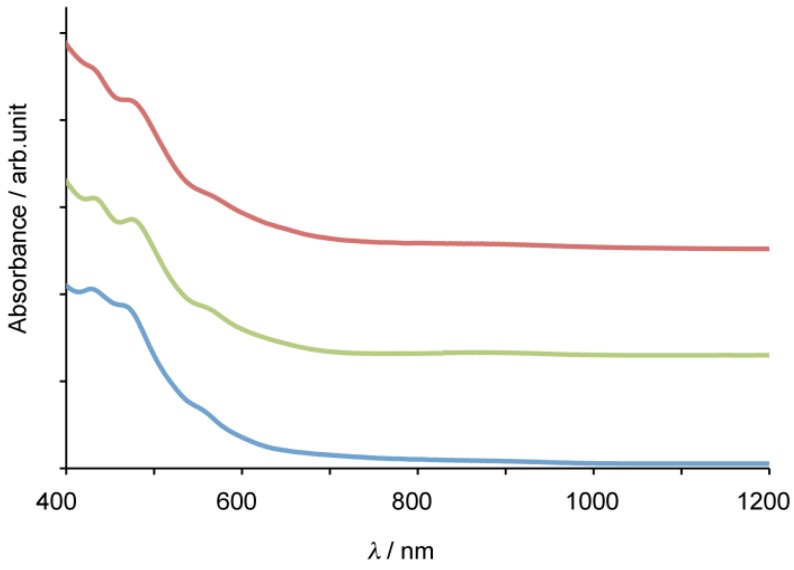
Vis/NIR absorption spectra of **9** (red), La_2_@*I_h_*-C_80_(Ad) (green), and La_2_@*I_h_*-C_80_ (blue) in CS_2_.

The absence of the cross peaks is reasonable because of the fact that the dihedral angle between H_4_ and C_2_ is close to 90°, leading to the coupling constant of zero based on Karplus equation [39,40,41]. It is noteworthy that the chemical shifts of the bonded cage carbons (C_1_ and C_2_, or C_1_′ and C_2_′) of **9** closely resemble those of the bonded cage carbons of La_2_@*I_h_*-C_80_(Ad) having [6,6]-open form [21]. Therefore, we concluded that the two diastereomers of **9** are associated with isomers A and B. Positive evidence of the possession of the [6,6]-open form is also provided by the similarity in the absorption spectra of **9** and La_2_@*I_h_*-C_80_(Ad). As shown in Figure 4, the absorption spectrum of **9** resembles those of La_2_@*I_h_*-C_80_ and La_2_@*I_h_*-C_80_(Ad), demonstrating that the intrinsic electronic structure of La_2_@*I_h_*-C_80_ is only slightly altered by the carbene addition.

To characterize the electrochemical properties, cyclic voltammetry (CV) and differential pulse voltammetry (DPV) were performed as shown in Figure S4 in the Supporting Information. It is reasonable to consider that the two diastereomers of **9** have identical redox potentials because the stereochemistry does not affect the electronic structure of La_2_@*I_h_*-C_80_[42]. Therefore, we assume that the waves of two diastereomers are entirely overlapped. As presented in Table 1, the first reduction potential of **9** is only shifted cathodically to 40 mV as compared to pristine La_2_@*I_h_*-C_80_. This trend is similar to the electrochemical behavior of La_2_@*I_h_*-C_80_(Ad) [21]. Results indicate that introduction of a glucopyranose moiety decreases the electron-accepting property because of the inductive effect. However, other reduction and oxidation waves were not identified because **9** was decomposed gradually during electrochemical measurements. Separation of the two diastereomers and deprotection of the glucopyranose moieties are currently under investigation.

**Table 1:** Redox potentials of La_2_@*I_h_*-C_80_ and its derivatives *^a^.*

compound	^ox^*E*_1_	^red^*E*_1_
**9**		–0.35
La_2_@*I_h_*-C_80_(Ad) *^b^*	0.49	–0.36
La_2_@*I_h_*-C_80_*^c^*	0.56	–0.31

*^a^* Values are in volts relative to Fc/Fc^+^ couple and obtained by DPV. *^b^* Data from ref. [21]. *^c^* Data from ref. [42].

## 3. Experimental

### 3.1. General

Toluene was distilled over benzophenone sodium ketyl under an argon atmosphere before use for the reactions. 1,2-Dichlorobenzene (ODCB) was distilled over P_2_O_5_ under vacuum before use. CS_2_ was distilled over P_2_O_5_ under an argon atmosphere before use. High-performance liquid chromatography (HPLC) isolation was performed using a recycling preparative HPLC system (LC-908; Japan Analytical Industry Co., Ltd.) and monitored by ultraviolet (UV) absorption at 330 nm. Toluene was used as the eluent. Mass spectrometry (Biflex III; Bruker Analytik GmbH) was performed with 9-nitroanthracene as matrix. The Vis/NIR absorption spectra were measured in a CS_2_ solution using a spectrophotometer (UV-3150; Shimadzu Corp.). Circular dichroism (CD) spectra were recorded on a spectropolarimeter (J-720W; Jasco Corp.). CD: scanning mode, continuous; scanning speed, 200 nm min^−1^; response, 2.0 s; bandwidth, 1.0 nm. Cyclic voltammograms (CVs) and differential pulse voltammograms (DPVs) were recorded on a BAS CV50W electrochemical analyzer. Platinum wires were used, respectively, as the working electrode and the counter electrode. The reference electrode was a saturated calomel reference electrode (SCE) filled with 0.1 M (*n*Bu)_4_NPF_6_ in ODCB. All potentials were referenced to the ferrocene/ferrocenium couple (Fc/Fc^+^) as the standard. CV: scan rate, 20 mV s^−1^. DPV: pulse amplitude, 50 mV; pulse width, 50 ms; pulse period, 200 ms; scan rate, 20 mV s^−1^. NMR spectra were obtained using an AVANCE-300 or AVANCE-500 spectrometer (Bruker Analytik GmbH) with a CryoProbe system (Bruker Analytik GmbH).

### 3.2. Preparation of La_2_@I_h_-C_80_ Glycoconjugate (**9**)

To a solution of 1.0 mg (8.1 × 10^−4^ mmol) of La_2_@*I_h_*-C_80_ in 20 mL of toluene was added 4.4 mg (8.0 × 10^−3^ mmol) of **8** at 0 °C followed by consecutive freeze–pump–thaw cycles. The mixture was stirred at room temperature for 1 h. The yield of **9** was estimated as 62% based on consumption of La_2_@*I_h_*-C_80_. The solvent was removed under vacuum, and the residue was purified by HPLC using a Buckyprep column to give glycoconjugate **9** as a dark brown solid: ^1^H-NMR (500 MHz, CD_2_Cl_2_/CS_2_ 1:3, 303 K) *δ* 7.4–7.0 (m), 5.57 (d, 7.7 Hz), 5.36 (d, 10.5 Hz), 5.28 (d, 10.5 Hz), 5.26 (d, 7.7 Hz), 5.17 (d, 10.5 Hz), 5.13 (d, 10.5 Hz), 5.05 (d, 7.7 Hz), 5.00 (d, 10.5 Hz), 4.9–4.8 (m), 4.7–4.5 (m), 4.1–4.0 (m) ppm; ^13^C-NMR (125 MHz, CD_2_Cl_2_/CS_2_ 1:3, 303 K) *δ* 152.84 (q), 152.83 (q), 152.71 (q), 152.66 (q), 150.34 (q), 150.32 (q), 150.29 (q), 150.23 (q), 150.11 (q), 150.01 (q), 149.98 (q), 149.95 (q), 149.45 (q), 149.39 (q), 149.30 (q), 149.26 (q), 149.23 (q), 148.69 (q), 148.67 (q), 148.61 (q), 148.36 (q), 148.23 (q), 148.15 (q), 148.03 (q), 146.97 (q), 146.84 (q), 146.42 (q), 146.32 (q), 146.28 (q), 145.34 (q), 145.32 (q), 145.29 (q), 144.52 (q), 144.33 (q), 144.23 (q), 144.19 (q), 144.14 (q), 144.11 (q), 143.11 (q), 143.03 (q), 142.98 (q), 142.97 (q), 142.91 (q), 142.83 (q), 142.73 (q), 142.61 (q), 142.58 (q), 142.56 (q), 141.99 (q), 141.94 (q), 141.90 (q), 141.87 (q), 141.85 (q), 140.12 (q), 139.98 (q), 139.92 (q), 139.86 (q), 139.59 (q), 139.53 (q), 139.42 (q), 139.37 (q), 139.24 (q), 139.21 (q), 136.69 (q), 136.50 (q), 136.47 (q), 136.44 (q), 136.39 (q), 136.35 (q), 136.34 (q), 136.23 (q), 136.21 (q), 136.20 (q), 136.18 (q), 136.07 (q), 135.65 (q), 135.40 (q), 134.73 (q), 134.31 (q), 134.25 (q), 134.13 (q), 134.05 (q), 133.71 (q), 133.70 (q), 133.39 (q), 133.25 (q), 133.21 (q), 133.20 (q), 133.19 (q), 133.15 (q), 133.13 (q), 132.00 (q), 131.00 (q), 130.89 (q), 130.87 (q), 130.84 (q), 130.68 (q), 130.61 (q), 130.44 (q), 130.12 (q), 130.05 (q), 130.04 (q), 129.98 (q), 129.85 (q), 127.7–126.6 (Ph), 125.66 (q), 125.57 (q), 125.31 (q), 125.26 (q), 124.81 (q), 123.38 (q), 122.01 (q), 119.87 (q), 119.83 (q), 117.16 (q; C_2_), 115.75 (q; C_2_′), 104.16 (q; C_1_), 104.11 (q; C_1_′), 91.09 (q; C_3_′), 89.51 (q; C_3_), 82.01 (CH), 81.62 (CH), 81.43 (CH), 80.73 (CH), 77.02 (CH), 75.40 (CH), 75.01 (CH), 74.43 (CH_2_), 74.23 (CH_2_), 74.17 (CH_2_), 73.61 (CH_2_), 73.45 (CH_2_), 72.63 (CH_2_), 72.57 (CH_2_), 72.52 (CH_2_), 68.09 (CH_2_), 68.03 (CH_2_) ppm (q = quaternary carbon signal); ^139^La-NMR (84.8 MHz, CD_2_Cl_2_/CS_2_ 1:3, 290 K) *δ* –360.2 ppm; MALDI-TOF MS (negative mode) calcd. for C_114_H_34_O_5_La_2_ [*M*] 1760.05, found [*M*^–^] 1760.1.

## 4. Conclusions

The results of this study demonstrate clearly that addition of electrophilic carbene is a powerful means to functionalize EMFs. The glycosilydene carbene generated *in-situ* from the corresponding diazirine precursor is highly reactive toward La_2_@*I_h_*-C_80_ at room temperature to afford two inseparable diastereomers of the mono-adducts, which are the first example of EMF glycoconjugates. We believe that this work paves the way for development of functionalized EMFs for biological and pharmacological applications.

## Supplementary Materials

Supplementary materials can be accessed on: http://www.mdpi.com/1420-3049/16/11/9495/s1.
