# Supplementary material for: Synthesis of Endohedral Metallofullerene Glycoconjugates by Carbene Addition
Source: Molecules. 2011 Nov 14;16(11):9495–504. doi: 10.3390/molecules16119495 (PMC6264206; doi:10.3390/molecules16119495)
Supplement: Supplementary file 1 [file molecules-16-09495-s001.pdf]

Supplementary Materials

Synthesis of Endohedral Metallofullerene Glycoconjugates by Carbene Addition

Michio Yamada,[1] Chika I. Someya,[2] Tsukasa Nakahodo,[2] Yutaka Maeda,[1] Takahiro Tsuchiya,[2] and Takeshi Akasaka[2],*

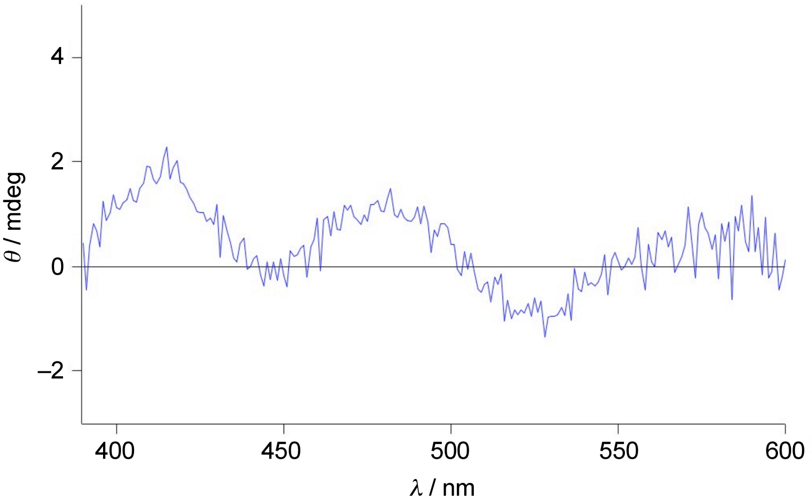

Figure S1. CD spectrum of **9** in CS_2.

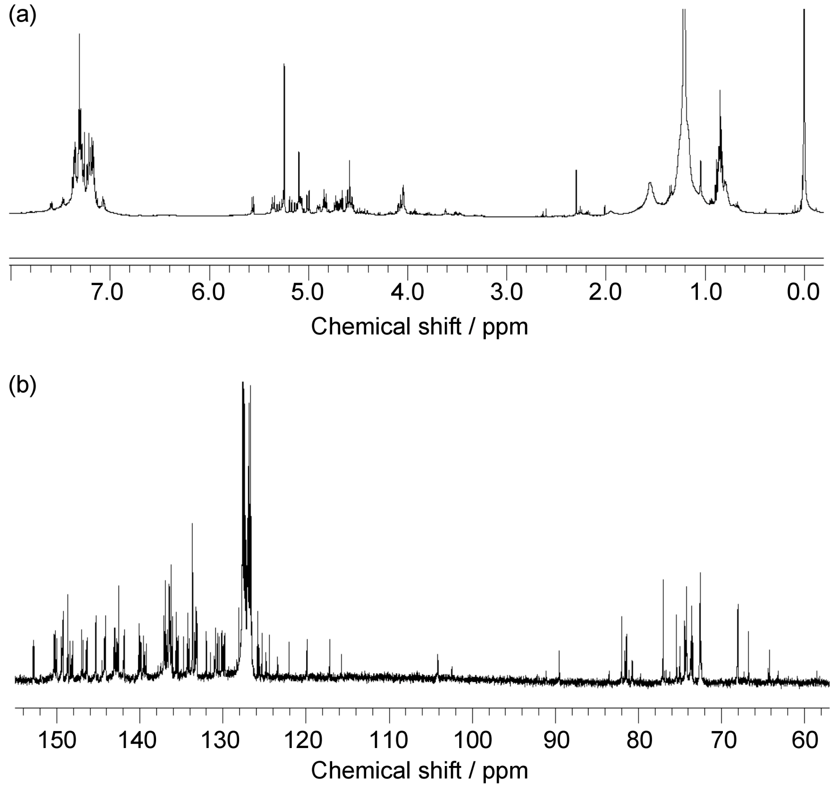

Figure S2. (a) 500 MHz ^{1}H and (b) 125 MHz ^{13}C NMR spectra of **9** in CD_2Cl_2/CS_2 (v/v 1:3) at 303 K.

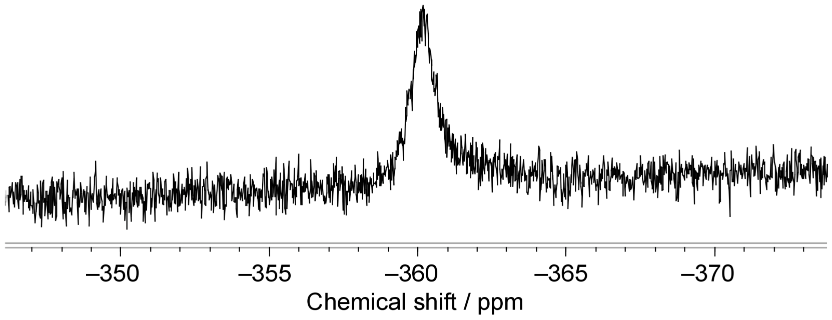

Figure S3. 84.8 MHz ^{139}La NMR spectrum of **9** in CD_2Cl_2/CS_2 (v/v 1:3) at 290 K.

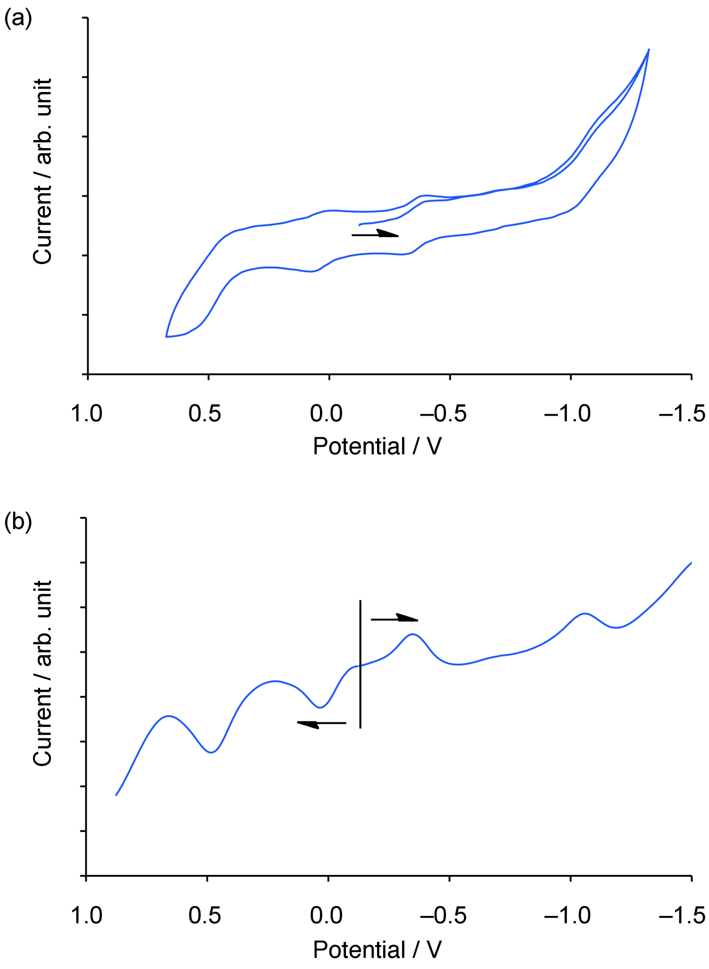

Figure S4. (a) CV and (b) DPV curves of **9** in ODCB containing 0.1 M (nBu)$_4$NPF$_6$.
